# Pioneering the Metaverse: The Role of the Metaverse in an Aging Population

**DOI:** 10.2196/40582

**Published:** 2023-01-20

**Authors:** Sara Shu, Benjamin K P Woo

**Affiliations:** 1 Department of Family Medicine Mayo Clinic Rochester, CA United States; 2 University of California, Los Angeles Sylmar, CA United States; 3 Chinese American Health Promotion Laboratory Los Angeles, CA United States

**Keywords:** metaverse, older adult, aging in place, dementia, gerontology, geriatric, digital health, digital technology, computer generated, artificial intelligence, virtual reality, mixed reality, augmented reality, aging, mental health

## Abstract

Amid a worldwide pandemic in the setting of an era of rapidly developing technologies, we turn now to the novel and exciting endeavor of pioneering the metaverse. Described as the conglomeration of augmented reality, virtual reality, and artificial intelligence, the metaverse has widespread applications in multiple settings, including revolutionizing health care. It also holds the potential to transform geriatric medicine, introducing new dimensions through which we can prevent social isolation, encourage health and well-being, and offer a new dimension through which we manage chronic disease. Although it is still a futuristic and novel technology, the metaverse’s realization may indeed be closer than we think.

Since the introduction of the World Wide Web, possibilities and imaginations have proliferated and expanded, and they have revolutionized the dissemination and sharing of information, communication, and connectedness through social media, videoconferencing, and gaming platforms. The *metaverse*, a term symbolizing the intersection of physical, augmented, and virtual reality in a shared web-based arena, was originally coined from the 1992 sci-fi novel *Snow Crash* [1], and it has now eerily become a tangible reality of the near future.

Technologies over the years have included many advances in virtual reality, augmented reality, artificial intelligence, and now the metaverse. The ability for people’s avatars to interact in real time with each other, much like what we see in the gaming industry with games like *Roblox* (Roblox Corporation) and *Animal Crossing* (Nintendo Co, Ltd), will soon become prevalent on social media and videoconferencing platforms—a virtual teleportation into digital space that will enable the transcendence of space and time.

The surge in technology, further bolstered by the COVID-19 pandemic, has transformed our videoconferencing platforms to enable work-from-home meetings, medical appointments via telehealth, and education across all levels. In the scientific community, conferences turned to virtual reality, using surprisingly interactive and advanced technologies, thereby enabling personal interaction via avatars among professionals across the globe. Virtual workspaces are currently being prototyped by companies like Microsoft. Facebook’s recent announcement, in which they pledged their pursuit of such future capabilities and stated that they were officially changing their name to *Meta,* set the stage for the next generation of technology [2].

Already, many have surmised the role of the metaverse in transforming health care in cardiovascular medicine, spine surgery, gynecology, behavioral and mental health, and even dentistry [3-8]. Such a novel concept opens possibilities for far-reaching applications, including using the metaverse to transform care for the aging population. With so many now interested in pioneering the metaverse, we can also take part in exploring what the metaverse can implicate in the lives of the older adult population. Digital presence in a time when physical presence is becoming less common and more difficult, such as during the current COVID-19 pandemic, makes us wonder whether the metaverse can truly be used toward promoting a society of intergenerational connectedness.

The COVID-19 pandemic has certainly highlighted the impact of social isolation on psychosocial health not only in the older adult population but also across all ages [9]. Fostering socialization through the use of technologies during this time has been an all too welcome solution. Older adults already experience the highest rates of social isolation and loneliness [10]. Combating this by facilitating socialization among loved ones already helps to decrease one of the leading causes of mortality in this population. Videoconferencing technologies enable social interaction with loved ones across the globe. The ability to interact with avatars or digital twins of loved ones in a virtual space will ultimately enhance the notion of *presence* when otherwise physically impossible [11].

These so-called *digital twins* provide yet another way through which medical visits may be augmented in the metaverse. Essentially virtual representations using real-time data that enable the running of endless simulations and extend to the human being, digital twins have the potential to guide disease management and become a tool for personalized and directed health care. Already, companies like Philips are developing technologies for creating digital twins of human hearts to help guide personalized medical decision-making and treatment [12]. Telehealth provides people living in remote areas or people who are otherwise unable to physically present to a clinic with the ability to carry out health care visits in virtual reality—another dimension that can enhance virtual diagnoses and care.

The use of such technologies can however prove challenging in older persons with reduced visual acuity, reduced manual dexterity, and cognitive impairment. Technological advancements have overcome some of these barriers, serving to improve functioning, tracking, and mobility, and have the potential to not only alleviate caregiver burden but also enable individuals to successfully age in place [13]. Alternately, there exist multiple forms of computerized cognitive training programs, or *serious games* (named as such due to their primary purpose being other than pure entertainment), that have been shown to help improve verbal, nonverbal, and working memory and therefore potentially have a role in slowing or preventing cognitive decline [14,15]. Incorporating cognitive training strategies is another application of the metaverse for this population.

The metaverse also holds the potential to address aspects of well-being, such as exercise and fitness. Much like the Wii Fit (Nintendo Co, Ltd) did for encouraging engaging in at-home workouts, nowadays apps like Supernatural (Within Unlimited, Inc) and FitXR (FitXR Limited) provide a means to exercise via virtual reality. Studies on virtual exercise via virtual reality applications and games have shown that these are viable modes of exercise that are able to elicit exercise intensities matching those in recommended guidelines and are being further studied to help inform developers about integrating exercise in the metaverse [16,17]. Integrating metaverse fitness into at-home exercises targeted toward older adults can help them to enhance their fitness in the comfort of their own homes. With the help of virtual reality, at-home exercise also can prove to be a means of helping someone to exercise when otherwise unable.

The first dementia villages were built in the Netherlands and Germany and introduced a novel concept of communal care, focusing on the psychological and emotional needs of those with dementia [18]. In the United States, the first dementia village—Glenner Town Square, San Diego, California—was built in 2018, using the setting of a 1950s town, and such villages are now being franchised and recreated around the country [19]. These villages build on the concept of reminiscence therapy and its potential benefit on cognition and mood in individuals with dementia [20]. Now, we can extend the creation of dementia villages into the metaverse by creating a digital “virtual playground,” so to speak. Individuals would be able to transcend time by going back to any time and any town of their choosing and enjoy the things with which they are familiar. Such reminiscence therapy can help individuals with dementia age with dignity by allowing them to find comfort in the memories of their past.

Through the metaverse, loved ones separated by physical distance may be able to interact within a virtual space in real time. Perhaps soon we will be able to create virtual spaces filled with specific memories of our own childhoods and store these memories to be able to share them with our future generations, so that they may be able to catch a glimpse of the world in which we once lived. As Amazon recently announced its current endeavor to develop artificial intelligence technology to enable Amazon Alexa to mimic voices of deceased loved ones, the capability to preserve or leave behind a piece of what was lost hints at the endless innovations to come [21]. The thought of this all too attainable future sounds like an episode from *Black Mirror*. Indeed, we are finding ourselves in a “digital catch-22” in which the potential benefits of virtual reality, augmented reality, and artificial intelligence technologies are ultimately dependent on having the desire to use these technologies and the knowledge and access to do so [22].

In pioneering the metaverse, future studies should focus on amplifying the potential of this virtual world in directly improving the mental health, and even indirectly improving the physical health, of the aging population. With the current paucity of evidence and developed technologies, there exists an urgency to invest resources to develop and apply such technologies for the care and well-being of our aging population. A once far-fetched, science-fictional imagination is now within our grasp.
